# The Paradoxical Protective Effect of Liver Steatosis on Severity and Functional Outcome of Ischemic Stroke

**DOI:** 10.3389/fneur.2019.00375

**Published:** 2019-04-12

**Authors:** Minyoul Baik, Seung Up Kim, Hyo Suk Nam, Ji Hoe Heo, Young Dae Kim

**Affiliations:** ^1^Department of Neurology, Yonsei University College of Medicine, Seoul, South Korea; ^2^Department of Internal Medicine, Institute of Gastroenterology, Yonsei University College of Medicine, Seoul, South Korea; ^3^Yonsei Liver Center, Severance Hospital, Seoul, South Korea

**Keywords:** stroke, transient ischemic attack, non-alcoholic fatty liver disease, stroke severity, stroke functional outcome

## Abstract

**Background:** There is very limited information on the relationship between non-alcoholic fatty liver disease (NAFLD) and the severity or functional outcomes of ischemic stroke or transient ischemic stroke (TIA). We investigated the correlation between NAFLD and stroke outcomes.

**Methods:** NAFLD was assessed in 321 patients with first-ever acute ischemic stroke or TIA, who underwent transient elastography from January 2014 to December 2014. The association of liver steatosis with stroke severity, assessed using the National Institute of Health Stroke Scale (NIHSS), was investigated using robust regression analysis. We also compared the functional outcome at 90 days according to the presence or burden of liver steatosis.

**Results:** NAFLD was observed in 206 (64.2%) patients. Patients with NAFLD had less severe stroke (median NIHSS score 2 vs. 3, *P* = 0.012) and more favorable functional outcome at 90 days (85.3 vs. 70.5, *P* = 0.004). Patients with NAFLD were likely to have a 23.3% lower [95% confidence interval (CI), −39.2 to −3.2%, *P* = 0.026] NIHSS score and a 2.5-fold higher (95% CI, 1.08–5.67, *P* = 0.033) possibility of favorable functional outcome in multivariate analysis.

**Conclusions:** Our study shows that a higher burden of liver steatosis seems to be associated with less severe stroke and better functional outcome after ischemic stroke or TIA.

## Introduction

Non-alcoholic fatty liver disease (NAFLD) is a spectrum of diseases from simple steatosis to steatohepatitis with varying degree of fibrosis, and liver cirrhosis (1, 2). NAFLD is becoming the most common chronic liver disease worldwide including Korea, affecting approximately 25% of the general population (3, 4). NAFLD is closely associated with obesity, insulin resistance, and type 2 diabetes mellitus, and is even recognized as a manifestation of metabolic syndrome in the liver (5). Furthermore, NAFLD is known to be clearly associated with cardiovascular morbidity and mortality (6, 7), and liver fibrosis is especially considered the key determinant (7–11).

Ischemic stroke is also suggested to be associated with NAFLD, especially liver fibrosis (7, 10, 12). In the previous study, we found that there was 41.5% of patients with NAFLD in ischemic stroke patients (10), similar with previous reports (23.0–42.7%) from other countries (12–14). However, there is a paucity of studies attempting to find the association between NAFLD and the stroke severity or functional outcome after ischemic stroke (12, 14, 15). Some previous studies, which defined NAFLD as the presence of elevated aminotransferase levels without apparent causes (12, 14, 15), failed to show a statistically significant association between NAFLD and stroke outcomes (12, 15).

Therefore, we sought to assess the association between NAFLD and the severity or functional outcome in patents with ischemic stroke. In this study, the presence of NAFLD was based on transient elastography (TE), which is known to be a well-qualified method for the diagnosis of NAFLD and could separately evaluate the degree of liver steatosis and stiffness (16, 17).

## Materials and Methods

### Patients and Evaluation

This was a retrospective, hospital-based observational study of patients with acute ischemic stroke or transient ischemic stroke (TIA) who were prospectively registered in the Yonsei Stroke Registry. The registry enrolls patients with acute ischemic stroke or TIA within 7 days of stoke onset who are admitted to the Severance Stroke Center, Seoul, Korea (18). All patients were thoroughly evaluated and managed according to the stroke care protocol based on current stroke guidelines.

**Figure 1:**
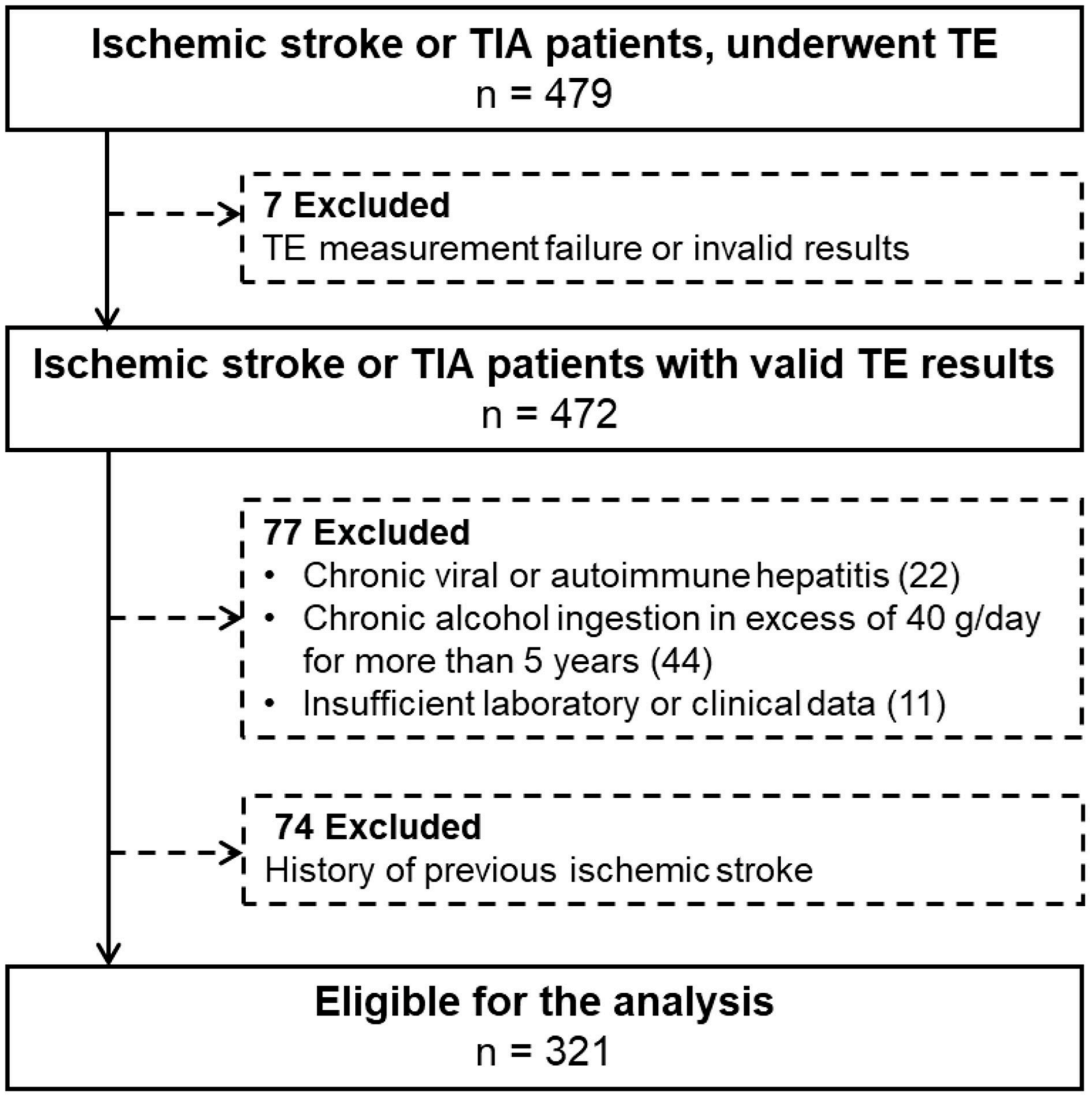
Patient selection.

Patients who were admitted from January 2014 to December 2014 and underwent TE as a protocol, were evaluated to investigate the association between NAFLD and ischemic stroke. Of the 479 included patients, patients with invalid or failed TE results, chronic viral or autoimmune hepatitis, heavy alcohol ingestion, and insufficient laboratory or clinical data were excluded. Additionally, we excluded 74 patients with a previous ischemic stroke. Finally, 321 patients were enrolled for this study (Figure 1).

This study was approved by the institutional review board of Severance Hospital, Yonsei University Health System. Informed consent was waived owing to the retrospective nature of the study.

### Liver Disease Evaluation

During the study period, all patients underwent TE examination. We used TE to evaluate liver parameters, instead of standard ultrasonography, because it is objective and reproducible method with a low inter-observer variability (19). Also, it expresses the degree of liver steatosis in a continuous manner as controlled attenuation parameter (CAP) and offer simultaneous measurements of liver stiffness (16, 17). The detailed methodology has been described in our previous studies (10, 20). The TE results for the degree of liver fibrosis were expressed as kilopascals (kPa) for liver stiffness (LS), and the degree of liver steatosis was expressed as CAP values. On the basis of previous studies, advanced liver fibrosis was defined as an LS value of >8.0 kPa (21, 22). For the presence of NAFLD based on CAP values, NAFLD was defined as a CAP value of >222 dB/m (23).

### Clinical Variables and Severity or Favorable Functional Outcome of Stroke

We collected the patients' data on vascular risk factors such as hypertension, diabetes, dyslipidemia, and body mass index (BMI, kg/m^2^); metabolic syndrome according to National Cholesterol Education Program Expert Panel and Adult Treatment Panel III criteria; current smoking; atrial fibrillation; and history of ischemic heart disease, ischemic stroke, or peripheral artery occlusive disease. We collected laboratory data including platelet count (× 10^3^/μL), aspartate aminotransferase (μkat/L), alanine aminotransferase (μkat/L), estimated glomerular filtration rate (mL·min^−1^·1.73 m^−2^), total cholesterol (mmol/L), triglycerides (mmol/L), high-density cholesterol (mmol/L), low-density cholesterol (mmol/L), and fasting blood glucose (mmol/L). Data on the duration (days) of hospitalization at the department of neurology were also collected. The subtype of stroke was determined based on Trial of ORG 10172 in Acute Stroke Treatment (TOAST) classification (24).

Stroke severity was assessed using the National Institute of Health Stroke Scale (NIHSS) by the stroke neurologists at admission. After discharge or transfer to other departments or institutionalized facilities, regular follow-up was implemented through routine outpatient clinic visits or telephone interviews. A favorable functional outcome at 90 days was defined as a modified Rankin scale score of 0–2 at 90 days after ischemic stroke or TIA.

### Statistical Analyses

Statistical and graphical analyses were performed using R version 3.4.2 (http://www.R-project.org). Univariate analysis was carried out with the independent *t*-test, or Mann-Whitney *U*-test for continuous variables and the χ^2^-test for categorical variables as appropriate. NIHSS was modeled as the natural logarithm of NIHSS plus 1 for skewness. Because of the lack of normality of the residuals for the NIHSS model, we used ordinary linear regression with robust standard errors to investigate the association between NIHSS score and liver steatosis, because residuals of the NIHSS model showed lack of normality. Determinants for favorable functional outcome were investigated using logistic regression analysis. Multivariate analysis was conducted with adjustments for age, sex, hypertension, diabetes mellitus, hypercholesterolemia, metabolic syndrome, BMI, and variables with *P* < 0.05 in univariate analysis. Finally, *P* < 0.05 was considered statistically significant.

## Results

### Baseline Characteristics

The baseline characteristics of the patients are presented in Table 1. Among 321 patients enrolled in this study, 206 (64.2%) patients had NAFLD based on the predetermined definition. Patients with NAFLD were more likely to be younger and to have hypertension; dyslipidemia; metabolic syndrome; no smoking habit; higher BMI, alanine aminotransferase level, or triglyceride level; and lower high-density lipoprotein level. There was no difference in the degree of liver fibrosis and distribution of strokes according to TOAST criteria between patients with and without NAFLD. Also, distribution of strokes according to TOAST criteria showed no difference according to whether patients were included or excluded (*p* > 0.05, data not shown).

**Table 1:** Demographic characteristics of study patients according to the presence of NAFLD.

	**Total (*n* = 321)**	**NAFLD (–) (*n* = 115)**	**NAFLD (+) (*n* = 206)**	***P-*value**
**DEMOGRAPHIC VARIABLES**				
Age, years	67.0 [56.0 to 74.0]	70.0 [58.5 to 76.0]	66.0 [56.0 to 73.0]	0.011
Sex, men	191 (59.5)	66 (57.4)	125 (60.7)	0.648
Hypertension	232 (72.3)	74 (64.3)	158 (76.7)	0.025
Diabetes	105 (32.7)	33 (28.7)	72 (35.0)	0.307
Dyslipidemia	56 (17.4)	13 (11.3)	43 (20.9)	0.044
Metabolic syndrome	166 (51.7)	44 (38.3)	122 (59.2)	< 0.001
Body mass index, kg/m^2^	23.6 [21.8 to 25.8]	21.8 [20.0 to 23.3]	24.6 [22.9 to 26.8]	< 0.001
Smoker	77 (24.0)	36 (31.3)	41 (19.9)	0.031
Ischemic heart disease	118 (36.8)	40 (34.8)	78 (37.9)	0.668
Peripheral artery occlusive disease	56 (17.4)	21 (18.3)	35 (17.0)	0.893
Atrial fibrillation	27 (8.4)	14 (12.2)	13 (6.3)	0.108
**STROKE VARIABLES**				
NIHSS at admission	2.0 [1.0 to 5.0]	3.0 [1.0 to 6.0]	2.0 [0.0 to 4.0]	0.012
TOAST classification (*n* = 290)				0.364
Cardioembolism	107 (36.9)	43 (40.2)	64 (35.0)	
Large artery atherosclerosis	39 (13.4)	11 (10.3)	28 (15.3)	
Lacunar	13 (4.5)	5 (4.7)	8 (4.4)	
Other determined etiology	18 (6.2)	3 (2.8)	15 (8.2)	
Negative evaluation	37 (12.8)	14 (13.1)	23 (12.6)	
More than two causes	76 (26.2)	31 (29.0)	45 (24.6)	
Duration of hospitalization	6.0 [5.0 to 9.0]	7.0 [5.0 to 10.0]	6.0 [4.0 to 8.0]	0.020
Favorable mRS at 90 days (*n* = 289)	231 (79.9)	74 (70.5)	157 (85.3)	0.004
**LABORATORY VARIABLES**				
Aspartate aminotransferase, μkat/L	0.4 [0.3 to 0.5]	0.4 [0.3 to 0.5]	0.4 [0.3 to 0.5]	0.441
Alanine aminotransferase, μkat/L	0.3 [0.2 to 0.4]	0.3 [0.2 to 0.3]	0.3 [0.2 to 0.5]	< 0.001
Platelet count, × 10^3^/mm^3^	229.0 [192.0 to 275.0]	222.0 [181.0 to 276.0]	237.0 [197.0 to 275.0]	0.221
Fasting blood glucose, mmol/L	5.8 [5.2 to 7.1]	5.6 [5.0 to 7.2]	5.9 [−5.2 to 7.0]	0.193
eGFR, mL·min^−1^·1.73 m^−2^	89.0 [76.0 to 90.0]	90.0 [80.0 to 90.0]	88.5 [75.0 to 90.0]	0.215
Total cholesterol, mmol/L	4.3 [3.7 to 5.0]	4.2 [3.6 to 4.9]	4.4 [3.7 to 5.1]	0.260
Triglyceride, mmol/L	1.2 [0.8 to 1.6]	1.0 [0.7 to 1.4]	1.3 [−0.9 to 1.7]	< 0.001
High-density lipoprotein, mmol/L	1.1 [0.9 to 1.3]	1.1 [1.0 to 1.3]	1.0 [0.9 to 1.2]	0.009
Low-density lipoprotein, mmol/L	2.6 [2.0 to 3.3]	2.5 [2.0 to 3.0]	2.6 [2.0 to 3.4]	0.288
**LIVER VARIABLES**				
Liver stiffness, kPa	4.7 [4.0 to 6.1]	4.6 [4.0 to 5.8]	4.7 [4.0 to 6.3]	0.750
Advanced fibrosis, >8 kPa	32 (10.0)	11 (9.6)	21 (10.2)	>0.999
CAP, dB/m	241.0 [207.0 to 275.0]	197.0 [177.0 to 209.5]	261.0 [242.0 to 289.0]	< 0.001

*NAFLD, non-alcoholic fatty liver disease; NIHSS, National Institute of Health Stroke Scale; TOAST, Trial of ORG 10172 in Acute Stroke Treatment; mRS, modified Rankin scale; eGFR, estimated glomerular filtration rate; CAP, controlled attenuation parameter. Data are given as median [interquartile range] or number (%)*.

### Stroke Severity and NAFLD

**Figure 2:**
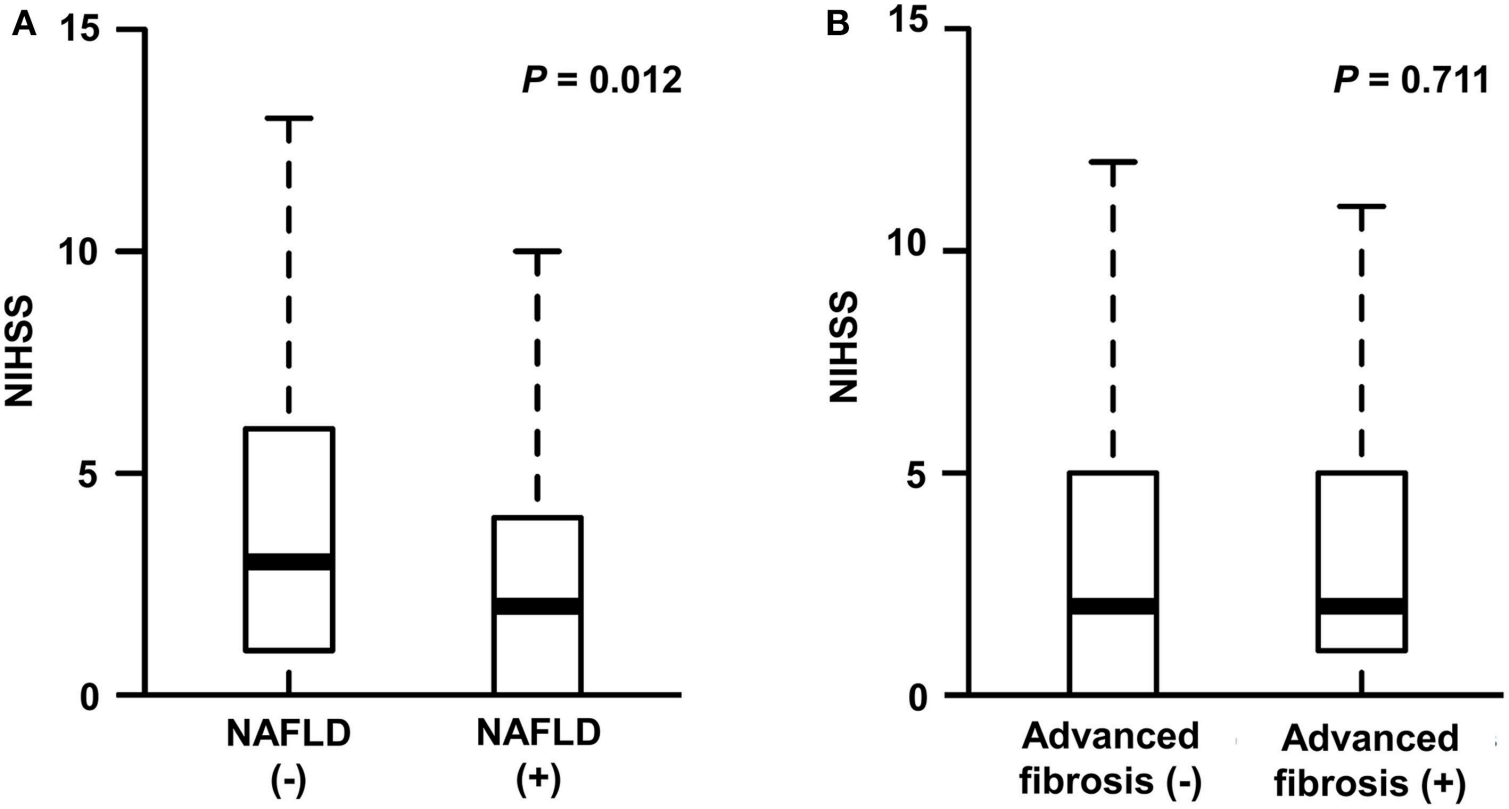
Stroke severity according to the presence of NAFLD **(A)** or advanced fibrosis **(B)**. NIHSS, National Institute of Health Stroke Scale; NAFLD, non-alcoholic fatty liver disease.

The median NIHSS score was 2 (interquartile range [IQR], 1–5). There were differences in NIHSS scores between patients with NAFLD and those without (median [IQR], 2 [0–4] vs. 3 [1–6], *P* = 0.012; Figure 2A). Patients with NAFLD had a shorter duration of hospitalization than those without (median [IQR], 6 [4–8] vs. 7 [5–10], *P* = 0.02; Table 1). However, the NIHSS score did not differ according to the presence of advanced fibrosis (*P* = 0.711, Figure 2B). In univariate robust linear regression analysis, the NIHSS score was associated with the burden of liver steatosis (*P* = 0.022) or NAFLD (*P* = 0.012), along with diabetes (*P* = 0.023), atrial fibrillation (*P* = 0.036), or peripheral artery occlusive disease (*P* = 0.032) (Table 2). In multivariate analysis, patients with NAFLD showed a 23.3% lower [95% confidence interval (CI) −39.2 to −3.2%, *P* = 0.026] NIHSS score than patients without NAFLD (Table 3). When parameters were used as continuous variables, such association was consistently noted (*P* = 0.039, Table 3). Advanced fibrosis or the burden of LS was not significantly associated with stroke severity (*P* > 0.05, Table 3).

**Table 2:** Univariate regression analysis.

	**NIHSS (*****n*** **=** **321)**		**Favorable functional outcome (*****n*** **=** **289)**	
	**Estimate (95% CI)**	***P*-value**	**Estimate (95% CI)**	***P*-value**
**DEMOGRAPHIC VARIABLES**				
Age, years	0.3% (−0.5 to 1.1%)	0.416	0.93 (0.91 to 0.96)	< 0.001
Sex, men	11.1% (−18.6 to 22.8%)	0.996	1.90 (1.06 to 3.41)	0.030
Hypertension	−7.2% (−26 to 16.3%)	0.516	0.33 (0.14 to 0.70)	0.007
Diabetes	28.4% (3.6 to 59.1%)	0.023	0.62 (0.34 to 1.12)	0.109
Dyslipidemia	6.9% (−18 to 39.5%)	0.621	1.39 (0.64 to 3.36)	0.431
Metabolic syndrome	4.2% (−14.8 to 27.3%)	0.689	0.68 (0.37 to 1.21)	0.188
Body mass index, kg/m^2^	−2% (−4.8 to 0.9%)	0.174	1.06 (0.98 to 1.16)	0.162
Smoker	21.9% (−3.5 to 53.8%)	0.097	1.80 (0.89 to 3.96)	0.120
Atrial fibrillation	32.6% (2 to 72.3%)	0.036	0.51 (0.26 to 1.04)	0.057
Ischemic heart disease	−69.2% (−75 to −62.1%)	0.093	1.50 (0.82 to 2.82)	0.198
Peripheral artery occlusive disease	45.5% (1.5 to 108.6%)	0.042	0.47 (0.19 to 1.29)	0.122
**STROKE VARIABLES**				
NIHSS at admission			0.83 (0.77 to 0.88)	< 0.001
Duration of hospitalization (days)			0.79 (0.73 to 0.86)	< 0.001
**LABORATORY VARIABLES**				
Aspartate aminotransferase, μkat/L	−24.7% (−51.7 to 17.3%)	0.210	0.60 (0.19 to 2.15)	0.399
Alanine aminotransferase, μkat/L	−25.6% (−44.5 to −0.3%)	0.048	2.49 (0.86 to 11.18)	0.163
Platelet count, × 10^3^/mm^3^	0% (−0.1 to 0.1%)	0.819	1.00 (1.00 to 1.00)	0.725
Fasting blood glucose, mmol/L	4.7% (1.3 to 8.3%)	0.007	0.95 (0.86 to 1.04)	0.212
eGFR,	0.2% (−0.4 to 0.8%)	0.538	1.01 (0.99 to 1.02)	0.432
Total cholesterol, mmol/L	7.7% (−1.5 to 17.8%)	0.102	0.97 (0.75 to 1.26)	0.804
Triglyceride, mmol/L	−0.2% (−9.8 to 10.4%)	0.968	1.40 (0.96 to 2.24)	0.127
High-density lipoprotein, mmol/L	−6.1% (−33.6 to 32.7%)	0.721	1.02 (0.39 to 2.85)	0.967
Low-density lipoprotein, mmol/L	12.3% (1.2 to 24.7%)	0.030	0.89 (0.66 to 1.21)	0.443
**LIVER VARIABLES**				
Liver stiffness, kPa	1.6% (−1 to 4.2%)	0.236	0.96 (0.89 to 1.02)	0.159
Advanced fibrosis, >8 kPa	5.6% (−24.5 to 47.8%)	0.750	0.77 (0.32 to 2.03)	0.565
CAP, dB/m	−0.3% (−0.5 to 0%)	0.022	1.01 (1.01 to 1.02)	< 0.001
NAFLD, >220dB/m	−24.9% (−39.8 to −6.3%)	0.012	2.44 (1.36 to 4.40)	0.003

*NIHSS, National Institute of Health Stroke Scale; eGFR, estimated glomerular filtration rate; CAP, controlled attenuation parameter; NAFLD, non-alcoholic fatty liver disease. Data are given as hazard ratio (95% confidence interval)*.

**Table 3:** Multivariate regression analysis of the degree of liver fibrosis and steatosis.

	**NIHSS (*****n*** **=** **321)**		**Favorable functional outcome (*****n*** **=** **289)**	
	**Estimate (95% CI)**	***P*-value**	**Estimate (95% CI)**	***P-*value**
**PARAMETERS AS CONTINUOUS VARIABLES**				
Liver steatosis, per 1 dB/m	−0.3% (−0.5 to 0%)	0.039	1.01 (1 to 1.02)	0.026
Liver stiffness, kPa	1.4% (−1.4 to 4.3%)	0.319	0.96 (0.88 to 1.05)	0.341
**PARAMETERS AS CATEGORIAL VARIABLES**				
NAFLD, >220 dB/m	−23.3% (−39.2 to −3.2%)	0.026	2.45 (1.08 to 5.67)	0.033
Advanced fibrosis, >8 kPa	−2.9% (−31 to 36.8%)	0.868	0.71 (0.23 to 2.38)	0.559

*NIHSS, National Institute of Health Stroke Scale; NAFLD, non-alcoholic fatty liver disease. Adjusted for age, sex, hypertension, diabetes, dyslipidemia, metabolic syndrome, body mass index, and variables with p < 0.05 in univariate analysis. Data are given as hazard ratio (95% confidence interval)*.

### Favorable Functional Outcome at 90 Days and NAFLD

**Figure 3:**
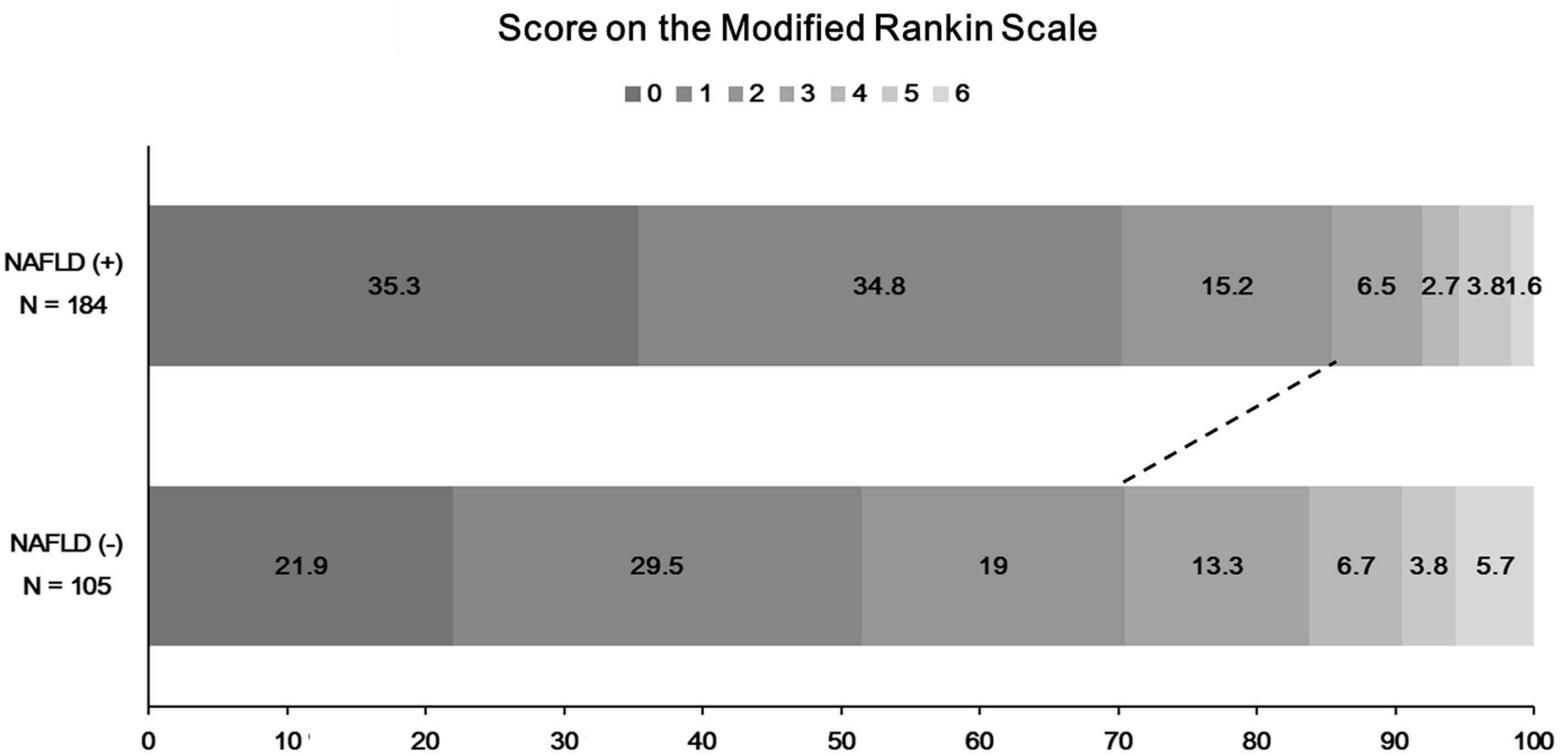
Distribution of functional outcome at 90 days according to the presence of NAFLD. A total of 32 patients without modified Rankin scale (mRS) scores in 90-day data were excluded. NAFLD, non-alcoholic fatty liver disease; mRS, modified Rankin scale.

The functional outcome at 90 days could be assessed among 289 patients. Patients with NAFLD were more likely to have a favorable functional outcome at 90 days than those without (157 [85.3%] vs. 74 [70.5%], *P* = 0.004; Figure 3). In univariate logistic regression analysis, age, male sex, hypertension, NIHSS score, duration of hospitalization, steatosis, or NAFLD was associated with a favorable functional outcome at 90 days (Table 2). In multivariate analysis, patients with NAFLD showed a 2.5-fold higher (OR 2.45, 95% CI 1.08–5.67, *P* = 0.033) possibility of favorable functional outcome (Table 3). This association was consistent when parameters were used as continuous variables (*P* = 0.026, Table 3). Advanced fibrosis or the burden of LS showed no significant association with functional outcome (*P* > 0.05, Table 3).

## Discussion

In this study, we found that NAFLD, not advanced fibrosis, was an independent predictor of less severe stroke and more favorable functional outcome at 90 days in patients with first-ever ischemic stroke or TIA. Those associations were consistent even after adjustment for a variety of known vascular risk factors, and even when liver parameters were used as continuous variables.

NAFLD is not a single disease entity but a spectrum of diseases, including simple fatty liver, steatohepatitis with fibrosis, and liver cirrhosis (1, 2). Previous studies evaluating the significance of liver fibrosis or steatosis suggested that liver fibrosis, rather than NAFLD, might act as a key prognostic factor for long-term clinical outcome (7–9, 11). Hepatic stellate cells, which play a critical role in liver inflammation and fibrogenesis, have been known to involve the elevation of circulating levels of several inflammatory, procoagulant, and oxidative stress markers, and to cause poor vascular outcomes (25). In our previous studies, we also indicated that advanced liver fibrosis might be a predictor of the occurrence of ischemic stroke and long-term outcomes of ischemic stroke patients, whereas isolated liver steatosis was not (10, 11). However, this study implied that stroke severity was dependent on liver steatosis, not on liver fibrosis, in patients without a previous ischemic stroke or TIA.

Considering the potential detrimental effect of NAFLD on the cardiovascular or cerebrovascular system (6, 7), the presence of NAFLD could be expected to lead to worse stroke profiles. However, in terms of the impact of NAFLD on stroke severity or functional outcome after stroke, there are only a few reports, which showed contradictory results (12, 14, 15). NAFLD could be associated with severe stroke or neurological deterioration during hospitalization in a specific stroke population such as patients with brainstem infarction (14). Some other studies conducted in the overall stroke population showed that the presence of NAFLD had some detrimental or neutral effects on the stroke severity. However, in those studies, the stroke severity was compared without adjusting for any potential confounders (12), or with only a small number of patients with NAFLD (15). Furthermore, NAFLD was defined based on a relatively arbitrary definition, such as aminotransferase level above the upper limit, which also led to some different results (2, 12, 15, 17). In our analysis based on TE including the overall stroke population, the presence or burden of NAFLD was found to be associated with mild stroke or favorable functional outcome even after adjustment for potential confounding factors.

The exact mechanism of this paradoxical phenomenon is not clear. However, our results could be disputed because of the differences in baseline demographics. In our analysis, although patients with NAFLD were more likely to have co-morbidities such as hypertension, dyslipidemia, metabolic syndrome, or higher BMI etc., the proportion of significant factors affecting the NIHSS score, such as diabetes, atrial fibrillation, or peripheral arterial occlusive disease, was similar between the 2 groups, or even lower in patients with NAFLD than in those without NAFLD. Furthermore, in our study, patients with NAFLD were actually 4 years younger than patients without NAFLD and had higher BMI. Previously, the obesity paradox for BMI and the risk of stroke or stroke outcome was proposed (26). These imbalances of underlying co-morbidities could have caused inverse relationship between liver steatosis and systemic inflammatory damages and finally led to paradoxical protective effect of liver steatosis on severity and functional outcome of ischemic stroke. However, the association between NAFLD and favorable outcome persisted even after extensive covariate adjustment for all favorable baseline characteristics.

One limitation of our study is that the retrospective single-center design might have caused a selection bias. However, no patient selection for performing TE was conducted in our study cohort. Another possible limitation is that liver status was not evaluated using liver biopsy, which is a gold standard method for the evaluation of NAFLD (1, 2). Nevertheless, TE is a well-established method (16, 17), and there is limitation in conducting liver biopsy in patients with acute stroke within acute stroke period. One more limitation is that we did not evaluated inflammatory, procoagulant, and oxidative stress markers, which are well-known to connect liver pathology with vascular outcomes (25). By measuring these markers and investigate the association of liver parameters and stroke outcomes with them, we could reveal that the paradoxical protective effect of liver steatosis is just a bystander phenomenon or one of under-recognized cascades. Future prospective study with those markers might be warranted.

In conclusion, a higher burden of liver steatosis, NAFLD, seems to be associated with less severe stroke and better functional outcome after ischemic stroke or TIA. This paradoxical association may be causally related or a kind of an epiphenomenon. Further studies are needed to clarify the possible mechanism.

## Ethics Statement

This study was approved by the institutional review board of Severance Hospital, Yonsei University Health System. Informed consent was waived owing to the retrospective nature of the study.

## Author Contributions

MB and YK drafted and revised the manuscripts, participated in study concept and design, conducted the statistical analyses, analyzed, and interpreted the data. SK participated in study concept and design, data interpretation, and assisted in data collecting. HN and JH participated in the study design and made contribution in revising the manuscript.

### Conflict of Interest Statement

The authors declare that the research was conducted in the absence of any commercial or financial relationships that could be construed as a potential conflict of interest.

## References

[1] Rinella ME. Nonalcoholic fatty liver disease: a systematic review. JAMA. (2015) 313:2263–73. 10.1001/jama.2015.5370 26057287

[2] Chalasani N Younossi Z Lavine JE Charlton M Cusi K Rinella M. The diagnosis and management of nonalcoholic fatty liver disease: practice guidance from the American Association for the Study of Liver Diseases. Hepatology. (2018) 67:328–57. 10.1002/hep.29367 28714183

[3] Younossi ZM Koenig AB Abdelatif D Fazel Y Henry L Wymer M. Global epidemiology of nonalcoholic fatty liver disease-Meta-analytic assessment of prevalence, incidence, and outcomes. Hepatology. (2016) 64:73–84. 10.1002/hep.28431 26707365

[4] Jeong EH Jun DW Cho YK Choe YG Ryu S Lee SM. Regional prevalence of non-alcoholic fatty liver disease in Seoul and Gyeonggi-do, Korea. Clin Mol Hepatol. (2013) 19:266–72. 10.3350/cmh.2013.19.3.266 24133664 PMC3796676

[5] Marchesini G Bugianesi E Forlani G Cerrelli F Lenzi M Manini R. Nonalcoholic fatty liver, steatohepatitis, and the metabolic syndrome. Hepatology. (2003) 37:917–23. 10.1053/jhep.2003.50161 12668987

[6] Targher G Day CP Bonora E. Risk of cardiovascular disease in patients with nonalcoholic fatty liver disease. N Engl J Med. (2010) 363:1341–50. 10.1056/NEJMra0912063 20879883

[7] Targher G Byrne CD Lonardo A Zoppini G Barbui C. Non-alcoholic fatty liver disease and risk of incident cardiovascular disease: a meta-analysis. J Hepatol. (2016) 65:589–600. 10.1016/j.jhep.2016.05.013 27212244

[8] Ekstedt M Hagstrom H Nasr P Fredrikson M Stal P Kechagias S. Fibrosis stage is the strongest predictor for disease-specific mortality in NAFLD after up to 33 years of follow-up. Hepatology. (2015) 61:1547–54. 10.1002/hep.27368 25125077

[9] Angulo P Kleiner DE Dam-Larsen S Adams LA Bjornsson ES Charatcharoenwitthaya P. Liver fibrosis, but no other histologic features, is associated with long-term outcomes of patients with nonalcoholic fatty liver disease. Gastroenterology. (2015) 149:389–97.e10. 10.1053/j.gastro.2015.04.043 25935633 PMC4516664

[10] Kim SU Song D Heo JH Yoo J Kim BK Park JY. Liver fibrosis assessed with transient elastography is an independent risk factor for ischemic stroke. Atherosclerosis. (2017) 260:156–62. 10.1016/j.atherosclerosis.2017.02.005 28222857

[11] Baik M Kim SU Kang S Park HJ Nam HS Heo JH Liver fibrosis, not steatosis, Associates with Long-Term Outcomes in Ischaemic Stroke Patients. Cerebrovasc Dis. (2019) 47:32–9. 10.1159/000497069 30763931

[12] Abdeldyem SM Goda T Khodeir SA Abou Saif S Abd-Elsalam S. Nonalcoholic fatty liver disease in patients with acute ischemic stroke is associated with more severe stroke and worse outcome. J Clin Lipidol. (2017) 11:915–9. 10.1016/j.jacl.2017.04.115 28579247

[13] Alkagiet S Papagiannis A Tziomalos K. Associations between nonalcoholic fatty liver disease and ischemic stroke. World J Hepatol. (2018) 10:474–8. 10.4254/wjh.v10.i7.474 30079133 PMC6068844

[14] Li H Hu B Wei L Zhou L Zhang L Lin Y. Non-alcoholic fatty liver disease is associated with stroke severity and progression of brainstem infarctions. Eur J Neurol. (2018) 25:577–e34. 10.1111/ene.13556 29281159

[15] Tziomalos K Giampatzis V Bouziana SD Spanou M Papadopoulou M Pavlidis A. Association between nonalcoholic fatty liver disease and acute ischemic stroke severity and outcome. World J Hepatol. (2013) 5:621–6. 10.4254/wjh.v5.i11.621 24303090 PMC3847945

[16] Castera L Forns X Alberti A. Non-invasive evaluation of liver fibrosis using transient elastography. J Hepatol. (2008) 48:835–47. 10.1016/j.jhep.2008.02.008 18334275

[17] Musso G Gambino R Cassader M Pagano G. Meta-analysis: natural history of non-alcoholic fatty liver disease (NAFLD) and diagnostic accuracy of non-invasive tests for liver disease severity. Ann Med. (2011) 43:617–49. 10.3109/07853890.2010.518623 21039302

[18] Lee BI Nam HS Heo JH Kim DI. Yonsei stroke registry. Analysis of 1,000 patients with acute cerebral infarctions. Cerebrovasc Dis. (2001) 12:145–51. 10.1159/000047697 11641577

[19] Myers RP Pollett A Kirsch R Pomier-Layrargues G Beaton M Levstik M. Controlled Attenuation Parameter (CAP): a noninvasive method for the detection of hepatic steatosis based on transient elastography. Liver Int. (2012) 32:902–10. 10.1111/j.1478-3231.2012.02781.x 22435761

[20] Kim YD Song D Heo JH Kim SU Kim BK Park JY. Relationship between cerebral microbleeds and liver stiffness determined by transient elastography. PLoS ONE. (2015) 10:e0139227. 10.1371/journal.pone.0139227 26421848 PMC4589390

[21] Wong VW Vergniol J Wong GL Foucher J Chan HL Le Bail B. Diagnosis of fibrosis and cirrhosis using liver stiffness measurement in nonalcoholic fatty liver disease. Hepatology. (2010) 51:454–62. 10.1002/hep.23312 20101745

[22] Roulot D Costes JL Buyck JF Warzocha U Gambier N Czernichow S. Transient elastography as a screening tool for liver fibrosis and cirrhosis in a community-based population aged over 45 years. Gut. (2011) 60:977–84. 10.1136/gut.2010.221382 21068129

[23] Yilmaz Y Yesil A Gerin F Ergelen R Akin H Celikel CA. Detection of hepatic steatosis using the controlled attenuation parameter: a comparative study with liver biopsy. Scand J Gastroenterol. (2014) 49:611–6. 10.3109/00365521.2014.881548 24611771

[24] Adams HP Jr, Bendixen BH Kappelle LJ Biller J Love BB Gordon DL. Classification of subtype of acute ischemic stroke. Definitions for use in a multicenter clinical trial. TOAST. Trial of Org 10172 in acute stroke treatment. Stroke. (1993) 24:35–41. 10.1161/01.STR.24.1.35 7678184

[25] Bataller R Brenner DA. Liver fibrosis. J Clin Invest. (2005) 115:209–18. 10.1172/jci24282 15690074 PMC546435

[26] Andersen KK Olsen TS. The obesity paradox in stroke: lower mortality and lower risk of readmission for recurrent stroke in obese stroke patients. Int J Stroke. (2015) 10:99–104. 10.1111/ijs.12016 25635277

